# Comparison of line probe assay to BACTEC MGIT 960 system for susceptibility testing of first and second-line anti-tuberculosis drugs in a referral laboratory in South Africa

**DOI:** 10.1186/s12879-017-2898-3

**Published:** 2017-12-28

**Authors:** Nontuthuko E. Maningi, Lesibana A. Malinga, John F. Antiabong, Ruth M. Lekalakala, Nontombi M. Mbelle

**Affiliations:** 1 Department of Medical Microbiology, Faculty of Health Sciences, University of Pretoria, Private Bag X323, Arcadia, 0007 South Africa; 2 Tshwane Division, National Health Laboratory Services, Pretoria, South Africa; 3 Tuberculosis Platform, South African Medical Research Council, Pretoria, South Africa

**Keywords:** Drug-resistance, *Mycobacterium tuberculosis*, Line-probe assay, MGIT 960 system, Isoniazid, Rifampicin, Ethambutol, Ofloxacin, Kanamycin

## Abstract

**Background:**

The incidence of multidrug-resistant tuberculosis (MDR-TB) is increasing and the emergence of extensively drug-resistant tuberculosis (XDR-TB) is a major challenge. Controlling resistance, reducing transmission and improving treatment outcomes in MDR/XDR-TB patients is reliant on susceptibility testing. Susceptibility testing using phenotypic methods is labour intensive and time-consuming. Alternative methods, such as molecular assays are easier to perform and have a rapid turn-around time. The World Health Organization (WHO) has endorsed the use of line probe assays (LPAs) for first and second line diagnostic screening of MDR/XDR-TB.

**Methods:**

We compared the performance of LPAs to BACTEC MGIT 960 system for susceptibility testing of bacterial resistance to first-line drugs: rifampicin (RIF), isoniazid (INH), ethambutol (EMB), and second-line drugs ofloxacin (OFL) and kanamycin (KAN). One hundred (100) consecutive non-repeat *Mycobacterium tuberculosis* cultures, resistant to either INH or RIF or both, as identified by BACTEC MGIT 960 were tested. All isoniazid resistant cultures (*n* = 97) and RIF resistant cultures (*n* = 90) were processed with Genotype®MTBDRplus and Genotype®MTBDRsl line probe assays (LPAs). The agar proportion method was employed to further analyze discordant LPAs and the MGIT 960 isolates.

**Results:**

The Genotype ®MTBDR*plus* (version 2) sensitivity, specificity, PPV and NPV from culture isolates were as follows: RIF, 100%, 87.9, 58.3% and 100%; INH, 100%, 94.4%, 93.5% and 100%. The sensitivity, specificity PPV and NPV for Genotype ® MTBDR*sl* (version 1 and 2) from culture isolates were as follows: EMB, 60.0%, 89.2%, 68.2% and 85.3%; OFL, 100%, 91.4%, 56.2% and 100%; KAN, 100%, 97.7%, 60.0% and 100%. Line probe assay showed an excellent agreement (k = 0.93) for INH susceptibility testing when compared to MGIT 960 system while there was good agreement (k = 0.6–0.7) between both methods for RIF, OFL, KAN testing and moderate agreement for EMB (k = 0.5). A high RIF mono-resistance (MGIT 960 33/97 and LPA 43/97) was observed.

**Conclusion:**

LPAs are an efficient and reliable rapid molecular DST assay for rapid susceptibility screening of MDR and XDR-TB. Using LPAs in high MDR/XDR burden countries allows for appropriate and timely treatment, which will reduce transmission rates, morbidity and improve treatment outcomes in patients.

## Background

In 2015, 480,000 cases of MDR-TB were estimated globally, with 10,000 of those occurring in South Africa [1]. In South Africa, more than 70% of people infected with TB are living with HIV. Absent detection of drug-resistant TB may result in treatment failure and the development of extensively drug resistant TB (XDR-TB). Extensively drug resistant TB is MDR-TB that is also resistant to quinolones and one of the first line injectable drugs, including capreomycin, kanamycin and amikacin. XDR-TB has been reported in about 117 countries and globally, 9.5% of MDR-TB cases have developed XDR-TB. The dynamics of the TB epidemic are influenced by timeliness of diagnoses which affects the duration of infectiousness. The TB burden can only be reduced by speeding up detection rates and identifying treatment gaps [1].

Detection and treatment of MDR-TB and XDR-TB requires susceptibility testing to screen for resistance to specific antibiotics and utilizing results to design a treatment regimen. Phenotypic drug susceptibility testing (DST) requires the cultivation of samples in culture media and systematically testing sensitivity of the cultures to specific drugs [2]. Phenotypic testing is time consuming and prone to high contamination rate. Currently, BACTEC MGIT 960, a rapid liquid phenotypic DST method, is used in many laboratories to test for resistance to first-line and second-line drugs. The MGIT system automatically reports drug susceptibilities according to predefined algorithms 4 to 13 days after inoculation [3]. In 2008, WHO endorsed the use of the molecular test GenoType® MTBDR*plus* (Hain Lifescience, Nehren, Germany) for rapid detection of high-risk MDR-TB cases directly from specimens. The GenoType ® MTBDR*plus* test is a molecular PCR-based amplification and reverse blotting assay that employs specific probes hybridized to nitrocellulose strips to detect tuberculosis and its resistance to rifampicin (RIF) and isoniazid (INH) drugs. The assay detects mutations in the *rpo*B gene for RIF resistance, in the *kat*G gene for high-level INH resistance and in the *inh*A regulatory region gene for low-level INH resistance [3]. In May 2016, the WHO recommended use of second line probe DST assay (GenoType ® MTBDR*sl*). This molecular method detects resistance to second-line fluoroquinolone (FQ) and injectable drugs (SLID) as well as detecting XDR-TB [1]. The second line (version 1) assay also detects resistance to first line ethambutol (EMB) drug targeting mutations on codon 306 of the *emb*B gene.

Recently, the rapid diagnosis and treatment of drug-resistant TB has been emphasized [1]. Effective treatment of MDR-TB and XDR-TB is very costly, particularly in low income countries such as South Africa. Therefore, a sensitive and specific diagnostic tool is needed to initiate appropriate therapy and reduce the spread of multidrug-resistant *M. tuberculosis* strains. Substantial reduction in time to diagnosis, early commencement of appropriate therapy and the potential to prevent transmission of drug-resistant strains are major advantages of newer molecular methods. We compared the performance of molecular line probe assay to MGIT 960 system for the detection of resistance to first- and second-line drugs.

## Methods

### Study design

**Fig. 1:**
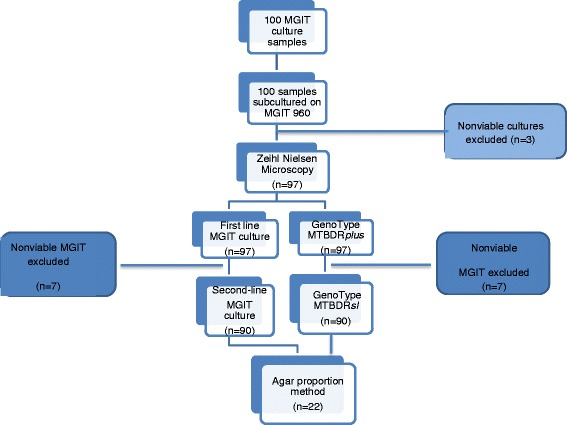
Flow diagram of MGIT 960 cultures for laboratory evaluation of LPA DST compared to MGIT 960 DST. MGIT-Mycobacterium Growth Indicator Tube, LPA-Line Probe Assay, ZN-Zeihl Nelson

This was a prospective descriptive study comparing the performance of the LPA (Hain Lifescience, Nehren, Germany) to BACTEC MGIT 960 system (Becton Dickinson Microbiology System, Sparks, MD, USA) for susceptibility testing of first and second-line anti-TB drugs. All testing was done in a routine diagnostic referral laboratory. We collected 100 consecutive non-repetitive *M. tuberculosis* cultures that were resistant to either isoniazid or rifampicin or both. Resistance was confirmed using the MGIT 960 system (Fig. 1). All samples were supplied by the National Health Laboratory Services, Tshwane Academic Division TB laboratory (NHLS/TAD) in the Department of Medical Microbiology University of Pretoria, South Africa. The NHLS/TAD is a high throughput diagnostic laboratory that receives specimens for microscopy, culture and drug susceptibility testing from surrounding Gauteng Province clinics and hospitals and other referring provinces such as Limpopo and Mpumalanga. The cultures were drawn from specimens of patients who were seeking care at primary facilities. The cultures used in the study were collected from January to June 2014. Agar proportion method was conducted at the South African Medical Research Council (SAMRC), Pretoria, TB Platform Unit, which is a Bio-safety level 2 laboratory. The HIV status of patients was not collected.

### MGIT subculture and DST

A total volume of 200 μl of MGIT 960 TB culture was inoculated in the MGIT 960 vials for sub culturing and growth detection. Positive cultures were stained using the Ziehl-Neelsen (ZN) staining method to confirm *M. tuberculosis*. Three isolates did not grow after subculture, 97 isolates were used for 1st line DST and 90 isolates were viable for 2nd line DST. Drug susceptibility tests for SM, INH, RIF, EMB, OFL and KAN, resistance were performed according to manufacturer’s instructions with the following drug concentrations: SM 1.0 μg/ml, INH 0.1 μg/ml, RIF 1.0 μg/ml, EMB 5.0 μg/ml, OFL 2 μg/ml and KAN 2.5 μg/ml. The instrument flagged the DST set complete when the growth control reached a growth unit (GU) value of 400. At that point, the GU values of drug-containing tubes were retrieved from the instrument by printing out the DST set report but interpreted manually for the second-line drugs.

### DNA isolation and line probe assay

For LPA DST, 97 isolates were used for the 1st line LPA and 90 isolates were viable after subculture for 2nd line LPA. DNA was isolated from liquid cultures using the Genolyse (R) Kit (Hain Lifescience, Germany) according to manufacturer’s instructions. Detection of first line and second line drug resistant TB was performed using the MTBDR*plus* and MTBDR*sl* (version 1 and 2) assays according to the instructions provided by the manufacturer (Hain Lifescience, Nehren, Germany). A volume of 5 μl of isolated DNA was used for amplification. All amplifications were performed under the same PCR conditions for culture and the primers utilized were provided by the LPA system.

### Agar proportion method

We ruled out discrepancies between LPA and MGIT liquid culture (Fig. 1) by conducting DST using the agar proportion method on the Microplate (Lasec, SA) from Lowenstein–Jensen medium culture. A suspension of 5–10 mg of drug resistant-TB culture was used to make a McFarland standard. An inoculum of 0.1 ml was delivered into each of the drug-free and drug-containing microplates. Each of the inoculated microplates were sealed with paraffin and incubated at 36 ± 1 °C. The inoculated plates were examined for contamination after 1 week of incubation and interpreted for drug susceptibility after four and 6 weeks of incubation. We assessed drug susceptibility visually by comparing the drug containing media (1:1 bacterial suspensions) and the drug-free control on which 1:100 bacterial suspensions were inoculated. The strain was reported resistant (R) if the drug-containing microplate had a growth of more than 1%.

### Quality control

*M. tuberculosis* H37Rv (ATCC 25177) was used as quality control (QC) for all methods, and included this strain in all the DST that was performed. This QC strain is susceptible to first- and second-line drugs tested in this study.

### Statistical analysis

All results were expressed in percentages. The agreement, sensitivity, specificity, positive and negative predictive values of LPA compared to BACTEC MGIT 960 system were calculated for INH, RIF, EMB, OFL and KAN. Agreement between the two methods was assessed using the kappa statistic. The kappa value was interpreted as: <0.2, poor; 0.21–0.4, fair; 0.41–0.6, moderate, 0.61–0.8, good and ≥0.81 excellent [4].

## Results

### First line drug susceptibility testing (DST)

A total of 100 cultures were analyzed in this study of which 97 were viable after subculture (Fig. 1). The results of the routine DST using the MGIT 960 system were compared to the results obtained by LPA. The MGIT 960 system detected 53/97 (54.64%) MDR-TB from which 15/97 (28.46%) were resistant to all first-line drugs tested (SIRE), 33/97 (34.02%) RIF mono-resistant and 11/97 (11.34%) INH mono-resistant. No streptomycin and EMB mono-resistance was observed. The line probe assay detected 40/97 (41.23%) MDR-TB and 43/97 (43.33%) RIF mono-resistant and 14/97 (14.43%) INH mono-resistant. The sensitivity, specificity, positive and negative predictive values for the detection of RIF resistance by LPA was found to be 100 (14/14), 87.9 (73/83), 58.3 (14/24) and 100 (73/73) percent respectively while for INH was 100 (43/43), 94.4(51/54), 93.5 (43/46) and 100 (51/51) percent respectively (Table 1).

**Table 1:** Performance characteristics of LPA for the detection RIF, INH, OFX, KAN and EMB

Drug	No of isolates	LPA + MGIT R	LPA + MGIT S	LPA R MGIT S	LPA S MGIT R	Sensitivity (%)	Specificity (%)	PPV (%)	NPV (%)	Agreement (%)	K-Value
^a^RIF	97	14	73	10	0	100 (76.8–100)	87.9(78.9–94.1)	58.3 (43.9–71.5)	100	89.7	0.68
^a^INH	97	43	51	3	0	100 (91.8–100)	94.4 (84.6–98.8)	93.5 (82.7–97.7)	100	96.9	0.93
^a^OFL	90	9	74	7	0	100 (66.3–100)	91.4 (83.0–96.4)	56.2(38.8–72.3)	100	92.2	0.68
^a^KAN	90	3	85	2	0	100 (29.2–100)	97.7(91.9–99.7)	60.0(27.60–85.5)	100	97.8	0.74
^a^EMB	90	15	58	7	10	60 (38.7–78.9)	89.2(79.06–95.56)	68.2(49.8–82.2)	85.3(78.1–90.4)	81.1	0.54

^a^ *RIF* Rifampicin, *INH* Isoniazid, *OFX* Ofloxacin, *KAN* Kanamycin, *EMB* Ethambutol, *S* susceptible, *R* resistant, *LPA* Line Probe Assay, *PPV* positive predictive value, *NPV* negative predictive value

Specific mutations were detected in 85.5% (83/97) RIF resistant isolates. Of these, 55.4% (46/83) had mutation in codon S531 L, 32.5% (27/83) in D516V, 8.43% (7/83) in H526Y and 3.6% (3/83) in H526D. Isoniazid mutations were detected in 55.6% (54/97) resistant isolates. Specific mutations in codon S315 T of *kat*G gene were found in 59.3% (32/54) of isolates. Mutations in *inh*A gene occurred in 40.7% (22/54) of INH resistant isolates of which 63.6% (14/22) had mutation in codon C-15 T and 36.4% (8/22) in T-8A (Table 2).

**Table 2:** Mutation pattern of *gyr*A, *gyr*B, *rrs, eis*, *rpo*B, *kat*G, *inh*A and *emb*B genes

Drug	Locus	Mutation	Frequency (no. of isolates)
OFX	*gyr*A	A90V	9
		S91P	1
		D94G	3
		D94A	1
		WT	2
	*gyr*B	–	–
KAN	*rrs*	A1401G	3
		C1402T	2
	*eis*	–	–
RIF	*rpo*B	S531 L	46
		D516V	27
		H526Y	7
		H526D	3
INH	*kat*G	S315 T1	32
		T8A	8
	*inh*A	C15T	14
EMB	*emb*B	M306 V	15
		M306I	9

*OFL* ofloxacin, *KAN* kanamycin, *RIF* rifampicin, *INH* isoniazid, *EMB* ethambutol

### Second line drug susceptibility testing

When performing second line DST, 90/100 MTB cultures were viable after subculture (Fig. 1). The results of DST using the MGIT 960 system were compared to those obtained by line probe assay. The MGIT 960 system showed 10% (9/90) OFL resistance and 3.3% (3/90) KAN resistance while LPA detected 17.7% (16/90) OFL and 5.5% (5/90) KAN resistance. Three isolates were detected as XDR-TB by the MGIT 960 assay and five isolates were identified as the XDR-TB by the line probe assay. The sensitivity, specificity, positive and negative predictive values for the detection of OFL by second line probe assay was 100 (9/9), 91.4 (74/81), 56.2 (7/16) and 100 (74/74) % respectively; for KAN was 100 (3/3), 97.7 (85/87), 60.0(2/5) and 100 (85/85) % respectively while for EMB it was 60 (15/25), 89.2 (5865), 68.2 (15/22) and 85.3 (58/68)% respectively using both version 1 and 2 assays (Table 1). Nine of the OFL resistant isolates had *gyr*A A90V mutation while five aminoglycoside resistant isolates had *rrs* A1401G and C1402T mutations (Table 2). There were no mutations detected on the *gyr*B and *eis* genes.

Line probe assay showed an excellent agreement (k = 0.93) for INH susceptibility testing when compared to MGIT 960 while there was good agreement (k = 0.6–0.7) between both methods for RIF, OFX and KAN. Agreement for EMB susceptibility was moderate (k = 0.5). Overall, concordance of RIF, INH, OFX, KAN and EMB results determined using LPA and MGIT 960 was found to be 89.7, 96.9, 92.2, 97.8 and 81.1% respectively (Table 1). There were 22 cultures that delivered discordant results for both first- and second-line drugs (3 resistant to INH, 10 to RIF, 2 to KAN and 7 to OFL). Isolates with discrepant results were resolved by repeating the DST with the agar proportion method. Agar proportion confirmed the original results with the MGIT 960 system while it confirmed the results with LPA in 13 cases (3 false-resistant for INH and 10 false-resistant to RIF). Moreover, the other nine discrepancies remain unchanged [OFL (*n* = 7) and KAN (*n* = 7)], with a high drug (2.0 μg/ml for OFX and 5.0 μg/ml) concentration, the discordance was overcome.

## Discussion

Recently, the rapid diagnosis of drug resistant-TB has received much attention. Drug resistant TB poses a great threat to TB control programs worldwide. Effective treatment of MDR-TB and XDR-TB is very expensive, particularly in low income countries such as South Africa. Early diagnosis and effective treatment requires a sensitive and specific diagnostic tool. According to the WHO, an effective treatment regimen depends on optimal susceptibility testing of *Mycobacterium tuberculosis* to anti-TB drugs [1]. The accuracy of susceptibility testing results varies with the drug tested as well as with the DST method.

In this study, we compared the performance of the MGIT 960 system to line probe assay for the detection of drug susceptibility to first and second line drugs. Line probe assays showed high sensitivity and specificity the detection of susceptibility to RIF (sensitivity-100% and specificity-87.9%) and INH (sensitivity-100% and specificity-94.4%). The excellent sensitivity (100%) for susceptibility to RIF and INH (this study) was higher than the pooled sensitivity (96.7%) reported by Nathavithara et al. in a recent meta-analysis of 74 studies using LPA in direct and indirect specimens [5]. We reported a slightly lower specificity for susceptibility to RIF (87.9%) and INH (94.4%) compared the same meta-analysis that reported a pooled specificity of 98.8 and 99.2% for RIF and INH respectively. The lower specificity of RIF can be explained by the “disputed” mutations H526Y (7 isolates) and H526D (3 isolates) which confer low level RIF resistance and are often classified as susceptible by phenotypic DST methods especially the MGIT 960 assay [6, 7]. These mutations are endemic in our setting and have been reported in significant numbers (8–20%) by researchers in South Africa and other countries [8–10]. The WHO recommends solid media for DST of RIF in strains harboring disputed mutations. We used the agar proportion method to resolve the discordance caused by the RIF resistance that was confirmed by LPA that identified these disputed mutations. Solid media DST is not practical in clinical settings, as results may only be available 4–6 weeks after culture, in comparison liquid culture DST takes 10 to 14 days. The disputed mutations have been implicated in poor treatment outcomes, therefore discordant RIF DST results should be seriously considered and confirmed using other rapid methods such as DNA sequencing. This will allow clinicians to design appropriate treatment in a timely matter. In resource poor settings that have many specimens for DST testing, confirmation of discordant results may be a challenge since new molecular rapid assays are not easily accessible and costly. In RIF resistant isolates, we observed missense mutations at codons 531, 516 and 526 of the *rpo*B gene. The S531 L missense mutation was most common in the *rpo*B gene accounting for 55.4% (46/83) of all RIF resistant isolates. This mutation pattern is similar to other studies [11–15]. High levels of RIF mono-resistance was confirmed by both MGIT 960 (33/97, 34%) and LPA (43/97, 44%) in this study. Increasing levels of RIF mono-resistant has been reported in South Africa at rates ranging from 8 to 13% [16–19]. In South Africa, RIF mono-resistance is increasingly observed in HIV-infected people [17]. Unfortunately, no data was collected on HIV status, limiting our interpretation of results. Due to the continuous increase in RIF mono-resistance in South Africa, RIF resistance is no longer a reliable marker for MDR-TB, making DST for the identification of MDR-TB even more important. In countries with high RIF mono-resistance, the use of Xpert MTB/RIF as a base-line rapid screening method for MDR-TB should be revised. This may avoid unnecessary exposure to other toxic TB drugs. We suggest DST using LPA from direct positive smear specimens in high burden countries. The disadvantage of LPA are longer turnaround times when high volumes of specimens need to be processed since the LPA requires separate DNA extraction before PCR and amplicon detection unlike the Xpert MTB/RIF assay that extracts, amplifies and detects resistance in real time.

Most frequently, mutations in the S315 T region can be attributed to INH high level resistance. We observed similar results, as 59.3% (32/54) of INH resistant strains had S315 T mutations in the *kat*G region. The *inh*A mutations occurred in 40.7% (22/54) of INH resistance isolates. INH promoter mutations are prevalent in South Africa and are associated with 40–60% and 80–90% of MDR and XDR-TB cases respectively [20]. Line probe assay is useful for the early detection of promoter mutations to guide treatment regimen in patients. We found 3 discordant INH resistance results (LPA vs MGIT 960) that were identified as false-resistance by the agar proportion method. Brossier et al., [21] reported that LPA may be more sensitive to the detection of low level INH resistance, but in this study, LPA did not detect some isolates with high level INH resistance. The WHO recommends that both the MGIT 960 system and line-probe assay be used for DST of first-line anti-TB drugs and that conventional solid-based DST be used to confirm MDR-TB [22].

Ethambutol is a first line drug used to treat susceptible TB. Although we detected no mono-resistance to ethambutol, second line probe assay methods detected resistance with a sensitivity and specificity of 63 and 89%. Other researchers have also reported sensitivity and specificity ranges between 68 to 87% [23, 24]. This low sensitivity and specificity highlights the need to identify targets responsible for EMB resistance and to improve the sensitivity of molecular diagnostic tests. Using MGIT may also identify a false susceptibility to EMB. In Bangladesh, Banu et al. [25] found that the MGIT system identified a false susceptibility to EMB in 49% of samples. Solid-based DST is recommended for such isolates. Currently a need exists to develop an alternative reliable rapid method for EMB susceptibility testing to replace phenotypic and LPA DST methods with low sensitivity and specificity.

In 2016, the WHO endorsed LPA for rapid screening of XDR-TB in MDR-TB patients. The variable sensitivity of this assay to different drugs means that this test should not be used to “rule out” resistance to second-line drugs but rather act as a rapid screening test for identifying second-line drug resistance, especially in suspected cases of XDR-TB. Rapid and reliable molecular assays for the detection of XDR-TB is critical in high burden MDR countries. We observed an excellent sensitivity and specificity of 100% and 91.4% for OFL and 100% and 97.7% for KAN respectively using LPA (version 1 and 2). The high sensitivity and specificity of LPA for identifying OFL resistance was similar to Gardee et al. [26] who reported a 100% and 98.5% sensitivity and specificity, however they reported a slightly lower sensitivity and specificity for KAN resistance (89.2% and 98.5% respectively). The sensitivity and specificity of LPAs conducted in low TB burden countries, such as Italy, is lower for both KAN (86% and 90%) and OFL (93% and 98%) resistance [27]. The LPA is expected to perform better in MDR high burden countries. We found a 100% concordance between LPA version 1 and version 2. Other reports have suggested that there was no significant improvement of the first line LPA assay after the addition of *gyr*B probes for the detection of OFL but a slight improvement after the addition of the *eis* promoter probes for the detection of aminoglycoside resistance. Unfortunately, seven cultures were non-viable for second line testing, meaning that the group included in the first line comparison (97 isolates) was slightly different than the group included in the second line comparison (90 isolates). This may bias the results, if the 7 lost strains were either highly resistant or highly susceptible. Agar proportion method failed to resolve discordant OFL and KAN results [OFL (*n* = 7) and KAN (*n* = 2)], however, with a high drug concentration (2.0 μg/ml for OFX and 5.0 μg/ml), the discordance was overcome.

We observed few cases of XDR-TB (5.5%). Compared to our study, the number of XDR-TB cases reported in other parts of South Africa such as the Western Cape, KwaZulu Natal and Eastern Cape Provinces is higher (14–26%) [28, 29]. Generally, the prevalence of XDR-TB is lower in the northern region of South Africa compared to the southern, eastern and the western regions. The reason for this variability is unknown but may be linked to the circulation of different genotypes.

Line probe assay results showed excellent agreement (k = 0.93) for INH susceptibility testing when compared to MGIT 960 system. There was good agreement (K = 0.6–0.7) between both methods for RIF, OFL, KAN and moderate agreement (k = 0.5) for EMB susceptibility. Overall, concordance of the LPA and MGIT 960 systems was RIF (89.5%), INH (96.9%), OFL (92.2%), KAN (97.8%) and EMB (81.1%). Similar concordance of 84.4–98.1% for RIF and 87.3–90.14% for INH between line probe assay and conventional DSTs have been reported [5, 30]. Discordant results between phenotypic DSTs and current rapid genotypic assays may not be due to all mutations conferring resistance to anti-TB drugs being included in rapid genotypic assays. Resistant mutations may result in variable (low, moderate or high) phenotypic expression of drug resistance. Silent mutations do occur at the genetic level with no change in drug susceptibility pattern. The LPA assay may miss silent or neutral mutations that result in phenotypical susceptibility. This may be an insignificant problem as such mutations may not affect treatment outcomes in patients. Silent or neutral mutations may also result in LPA detecting more resistant isolates compared to the phenotypic MGIT 960. Alternatively, MGIT 960 fails to detect low level resistance mutation below drug breakpoint, which may have evolutionary consequences.

The turnaround time for LPA DST from culture specimens takes 10 to 14 days. Other reports have shown that turnaround time can be reduced to 1 to 2 days when LPA is used on direct specimens. Meaza et al. [31] used LPA with high sensitivity and specificity of 96% and 100% respectively in sputum positive specimens in 2 days. The LPA is the only rapid molecular diagnostic assay that can detect both RIF and INH (MDR-TB) resistance simultaneously in a short period of time. We suggest that LPA be used as a baseline method for the rapid detection of MDR-TB in smear positives especially in high burden countries to improve treatment outcomes in patients. Reducing the time to diagnosis, commencing appropriate therapy timeously and preventing transmission of drug-resistant strains are major advantages of these newer molecular methods. In comparison to the MGIT 960 system, LPA is an accurate and time-efficient method for the detection of drug-resistance TB. However, proper laboratory design, standard biosafety procedures and quality control to avoid cross-contamination and reduce costs are critical for LPA in resource poor countries. While the MGIT 960 system is cheaper than the LPA, it is difficult to perform and requires high standards of biosafety.

Information presented by the LPA of all the currently available platforms increases the options available to initiate and optimize treatment for mono, poly and multi-drug resistant tuberculosis. The excellent agreement between the LPA and culture DST in the detection of low level INH resistant (*inh*A) is very important as it allows clinicians to increase the dose of INH for the drug to be effective and to exclude ethianomide in treatment regimen due to cross-resistance. The detection of high level INH resistance (*kat*G) therefore allows the drug not be an option.

### Limitations of the study

The first line DST results for ethambutol were compared with the 90 isolates used for second line DST. In this study, 7 isolates were non-viable for second line testing; therefore not all isolates included in the first line testing were tested for second line DST. The comparison of these assays was slightly biased. Also, agar proportion DST was only used for discordant isolates between the MGIT 960 and line probe assay and not for all cultures.

## Conclusion

We observed great diagnostic performance of the LPA, confirming its reliability and efficiency for the rapid diagnosis of MDR-TB and early detection of possible XDR-TB. The high sensitivity and specificity by the second line LPA for the detection of OFL and KAN suggest that it may achieve performance characteristics similar to those of the first line probe assays currently available for the detection of MDR-TB and its implementation for the screening of XDR-TB cases in settings already performing the MDR-TB assays would be advantageous.

## Abbreviations

DST: Drug susceptibility testing
EMB: Ethambutol
FQ: Fluoroquinolone
INH: Isoniazid
KAN: Kanamycin
LPA: Line probe assay
MDR-TB: Multidrug resistant tuberculosis
MGIT: Mycobacterium growth indicator tube
MRC: Medical Research Council
NHLS/TAD: National Health Laboratory Services/Tshwane Academic Division
NPV: Negative predictive value
OFL: Ofloxacin
PPV: Positive predictive value
QC: Quality control
RIF: Rifampicin
SM: Streptomycin
TB: Tuberculosis
WHO: World Health Organization
ZN: Ziehl-Neelsen
